# Optically Mapping Multiple Bacterial Genomes Simultaneously in a Single Run

**DOI:** 10.1371/journal.pone.0027085

**Published:** 2011-11-21

**Authors:** Matthew C. Riley, James Eric Lee, Emil Lesho, Benjamin C. Kirkup

**Affiliations:** 1 Walter Reed Army Institute of Research, Silver Spring, Maryland, United States of America; 2 Multidrug Resistant Organism Repository and Surveillance Network (MRSN), US Army, Silver Spring, Maryland, United States of America; 3 Uniformed Services University of the Health Sciences, Silver Spring, Maryland, United States of America; University of Georgia, United States of America

## Abstract

Optical mapping of bacterial chromosomes provides an unambiguous low-resolution sequence scaffold of the entire chromosome. In comparison to some techniques, such as pulse field gel electrophoresis, cost and throughput limit the application of this technique outside of genome finishing. We have demonstrated the production of multiple bacterial maps using a single set of consumables; this significantly reduces the time and expense of map production.

## Introduction

Optical mapping is increasingly used to characterize genomes [1], [2], [3]. The map is an ordered restriction fragment list tracking the physical structure of each replicon without amplification. This is accomplished by binding the DNA physically to a surface (referred to as the ‘mapcard’), cutting it at restriction endonuclease sites, and labeling it for visualization. The images are recorded and the length of the ordered fragments is calculated. Because DNA shears during the extraction process, optical mapping is not as simple as following a single chromosome; instead, fragments are assembled together in a process analogous to that used for genome assembly after sequencing. Maps can be used for finding large insertions, deletions, duplications and rearrangements otherwise difficult to characterize even with sequencing; and for scaffolding sequenced genomes without the use of numerous PCR reactions (for example, in conjunction with SOMA; www.cbcb.umd.edu/soma).

Service laboratories charge thousands of dollars for a single map. The instrumentation costs ∼$300,000, and the consumables are ∼$500 for each run. The time to image the DNA and assemble the map is also significant (∼8 per day, per instrument and technician). Simultaneous mapping of multiple genomes would reduce cost and labor while increasing throughput, almost proportionally to the number of maps produced from each run.

**Figure 1:**
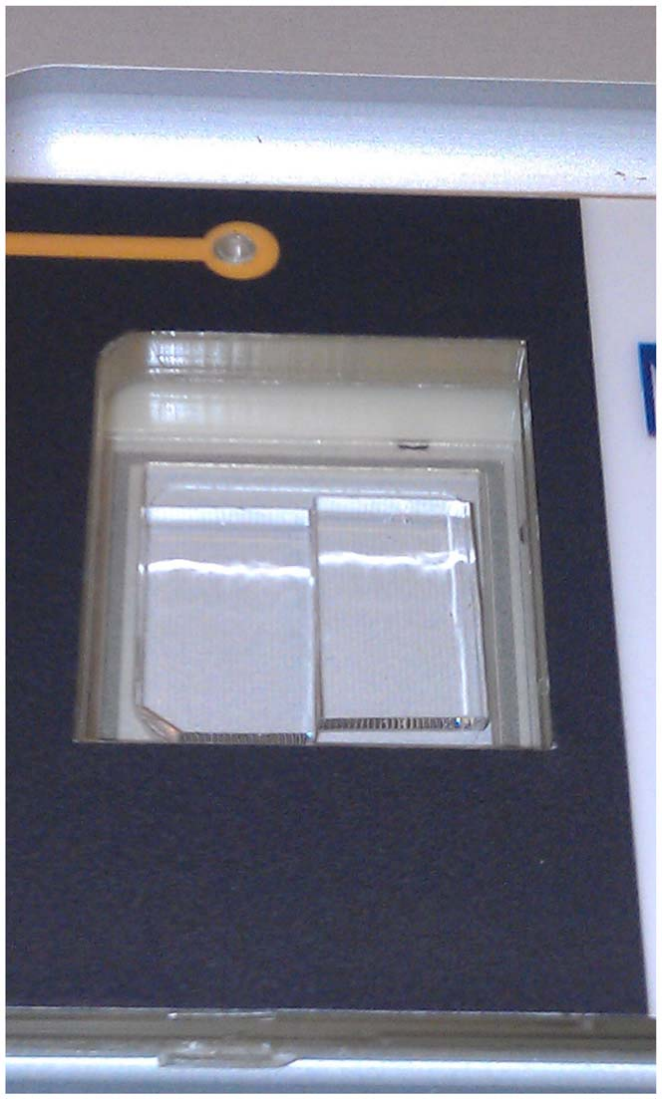
Multiple Surfaces. A surface subdivided in two permits parallel mapping efforts during a single run with a single set of consumables.

There are at least two ways to map multiple genomes on a single mapcard. One is to subdivide the mapcard physically; at least, the portion to which DNA adheres (Figure 1). This subdivides the surface area available for each DNA preparation but otherwise leaves the mapping process unaffected. The preparations are physically separated and do not interfere with each other. This method has been successfully implemented within our group but is limited by the physical ability to subdivide the surface; it seems limited to division in two at this time. It works well because the total capacity of the surface is often far beyond what is needed to assemble a robust bacterial genome map. The other strategy is to combine the DNA preparations prior to applying them to a single surface. This second method is the discussed at greater length for the remainder of this paper, as it does not have the genome limit of two the other method does. Also, there are implications for potentially mapping mixed unknown samples and resolving at least parts of each genome, if not full length, for identification. It has not escaped our notice that the methods can themselves be combined.

In the context of eukaryotic genomes, mapping multiple chromosomes simultaneously on the same mapcard is inevitably necessary because the DNA from the separate chromosomes cannot be segregated prior to mapping. The mapping of eukaryotic genomes has been accomplished many times. In the case of bacteria, the risk of failure has discouraged attempting more efficient mapping, particularly in closely related organisms. However, as presented here, mapping several bacterial genomes on a single mapcard is practical.

To successfully map multiple bacterial genomes on a single mapcard, the genomes being mapped must be amenable to processing with the same restriction endonuclease. This unavoidable restriction on the selection of genomes to be co-processed is not difficult to overcome. Compatible organisms can be selected in advance. It is this limitation that most clearly enforces the need for some upstream identification of the bacteria to be mapped.

Second, maps might become comingled during assembly of mixed DNA preparations. This would theoretically result from co-mapping genomes which share substantially identical sequence over long stretches. The question is: how long of a stretch, in what organizational pattern? Most stretches of shared homology are too short to cause any trouble. Typical molecule lengths are 150 Kb–1.5 Mb. Coverage depths exceed 40× (frequently 20 k–30 k molecules are imaged). In this context, two 5 Mb bacterial genomes will not be comingled unless they merge into a single genome within the limits of certainty for the detection of cut sites over the span of the longest molecules included in the assembly. Even if the assembled molecules were only 300 Kb in median length, continuous stretches of nearly ¼ the genome must be indistinguishable by mapping for concatenation to occur.

As a result, mapping genomes simultaneously will not result in ‘false’ maps unless two of the strains are nearly identical not just in base pair composition but also in sequence orientation. We have demonstrated mapping of *Shigella dysenteriae* and *Escherichia coli* simultaneously, despite their very close phylogenetic relationship (*Shigella* and *Escherichia coli* are generally considered to be within a single species, but are segregated at the genus level for historical reasons [4]); two clones of *Shigella* would likely not map together successfully using the mixed DNA method. Similarly, based on reference maps being generated at this time, genomes from distinct species of *Acinetobacter* should map simultaneously without ambiguity despite being within a single genus; but many clinical strains of *Acinetobacter baumannii*, distinguished by a few insertions, are too closely related to be mapped independently on the same mapcard.

A third concern is that the quality and condition of one DNA preparation could disrupt reading the entire mapcard; either because the preparation is contaminated or because it is too concentrated. In that second case, the ratio of the DNA preparations results in one map crowding out others. It should be noted that the risk here is merely a waste of material; there is no risk of erroneous data being accepted. Mapping typically involves a quality control step prior to preparing the mapcard. Normal quality control eliminates the risk of contaminating the mapcard or crowding out other genomes by failing poor DNA preparations and ensuring the DNA quality, concentration and length are optimal for a successful preparation; however, in an environment of routine successful DNA preparation, costly quality control steps might be bypassed and the risk of a failed DNA preparation considered acceptable. Ultimately, the combined mapping strategy is more cost and time effective than the single mapping strategy unless a high percentage (∼25%, if 3 strains are being mapped together routinely) of the DNA is bad, in which case DNA quality control should be reinstituted.

## Results and Discussion

**Figure 2:**
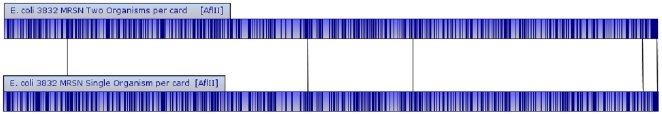
*E. coli* Genomes. Complete circularized *E. coli* genome maps created as a result of a mixed DNA mapcard run aligned to an identical map from individual run.

**Figure 3:**
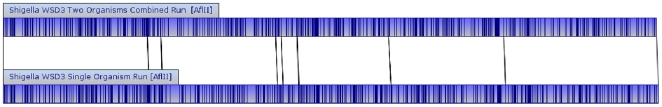
*Shigella* Genomes. Complete circularized *S. dysenteriae* genomes from mixed DNA mapcard run aligned to genome from individual run.

We have already mapped two bacterial genomes on a single mapcard using mixed DNA sample preparations (Figures 2 and 3). Additionally, two *Enterococcus* species were mapped on a single mapcard by cutting the channel forming device into two separate pieces; these have also been mapped independently and are 100% identical as well. Given the data produced from a single mapcard under ideal conditions, this method will permit up to ten bacterial genomes (5 Mb each) to be mapped on a single mapcard. However, the complexity of strain selection and the risks of mapcard failure steadily increase and the benefits diminish as the number of strains per mapcard increases. Three genomes per mapcard represents a substantial cost and time savings; however, the impact of these savings on operations depends on the economics of the laboratory as a whole. Our data (Table 1) indicate that under normal laboratory conditions, three to four strains per mapcard is a reasonable expectation when mixing DNA preparations. Concerns of lowered quality or depth of coverage are legitimate, but our work here shows (Table 2) it is possible to maintain within the generally accepted scaling factor and far above 40× minimum depth of coverage.

**Table 1:** Mapping Statistics.

	Mixed	*Escherichia*	*Shigella*
Total number of molecules:	12,482	7,009	9,804
Genomes Assembled:	2	1	1

These summary statistics list the number of molecules assembled to maps and the number of map assemblies generated during the processing of data from a single mapcard. The mixed mapcard bore DNA from both *Escherichia* and *Shigella*. The maps which were generated were circular and passed standard quality control.

**Table 2:** Assembly Statistics.

	Aligned maps	Length(Mb)	Avg. depth	Avg. Mol. Size(kb)
*E. coli* (combined)	1609	5.000	109	341
*E. coli* (single)	1045	5.160	77	382
*S. dysenteriae* (combined)	1589	4.176	111	293
*S. dysenteriae* (single)	1229	4.251	111	386

The columns display the values for the assembly statistics for E. coli and S. dysenteriae genomic maps from the combined run and individual runs. All genomes were circularized and passed standard QC.

The optimum number of maps on a single mapcard is a function of the operational and economic constraints of each laboratory. At this time, however, we do not foresee a significant gain beyond four maps on a single mapcard, which brings the consumables cost to $129 USD and increases the throughput of the entire process almost 4-fold, given that DNA preparations can be done in parallel. This modification makes mapping process more practical and amenable to incorporation into clinical and near-real time epidemiologic applications.

## Materials and Methods

An OpGen® Argus® system maintained under service contract was used to collect the data. The assemblies and figures were created with MapManager™ software. The currently recommended high molecular weight DNA extraction was performed, using magnetic bead technology developed by Agencourt Bioscience Corp®. The protocol provided by the manufacturer was followed unmodified for these samples, though certain organisms require tailored DNA preparation strategies. DNA extraction was performed independently on each strain. Purified DNA from the two strains was diluted to the appropriate concentration (approximately 5 molecules per image at or above ∼200 Kb). Each DNA sample was quality checked on a QCard, as was the combined DNA preparation.
